# *Cronobacter sakazakii* ATCC 29544 Autoaggregation Requires FliC Flagellation, Not Motility

**DOI:** 10.3389/fmicb.2017.00301

**Published:** 2017-02-28

**Authors:** Jennifer L. Hoeflinger, Michael J. Miller

**Affiliations:** Department of Food Science and Human Nutrition, University of Illinois at Urbana ChampaignUrbana, IL, USA

**Keywords:** autoaggregation, *Cronobacter sakazakii*, flagella, FliC, protein-protein interactions

## Abstract

*Cronobacter sakazakii* is an opportunistic nosocomial and foodborne pathogen that causes severe infections with high morbidity and mortality rates in neonates, the elderly, and immunocompromised individuals. Little is known about the pathogenesis mechanism of this pathogen and if there are any consequences of *C. sakazakii* colonization in healthy individuals. In this study, we characterized the mechanisms of autoaggregation in *C. sakazakii* ATCC 29544 (CS29544). Autoaggregation in CS29544 occurred rapidly, within 30 min, and proceeded to a maximum of 70%. Frameshift mutations in two flagellum proteins (FlhA and FliG) were identified in two nonautoaggregating CS29544 clonal variant isolates. Strategic gene knockouts were generated to determine if structurally intact and functional flagella were required for autoaggregation in CS29544. All structural knockouts (Δ*flhA*, Δ*fliG, and* Δ*fliC*) abolished autoaggregation, whereas the functional knockout (Δ*motAB*) did not prevent autoaggregation. Complementation with FliC (Δ*fliC*/cfliC) restored autoaggregation. Autoaggregation was also disrupted by the addition of exogenous wild-type CS29544 filaments in a dose-dependent manner. Finally, filament supercoils tethering neighboring wild-type CS29544 cells together were observed by transmission electron microscopy. *In silico* analyses suggest that direct interactions of neighboring CS29544 FliC filaments proceed by hydrophobic bonding between the externally exposed hypervariable regions of the CS29544 FliC flagellin protein. Further research is needed to confirm if flagella-mediated autoaggregation plays a prominent role in *C. sakazakii* pathogenesis.

## Introduction

*Cronobacter* spp. are motile, biofilm-forming, facultative anaerobic Gram-negative bacilli. *Cronobacter sakazakii*, formerly known as *Enterobacter sakazakii* (Iversen et al., 2008), the most prominent species, is an opportunistic pathogen associated with fatal infections in neonates and immunocompromised children and adults (Lai, 2001). Most notably, *C. sakazakii* infections in neonates have been linked epidemiologically to the consumption of powdered infant formula (PIF) (Biering et al., 1989; Simmons et al., 1989; van Acker et al., 2001). Furthermore, *C. sakazakii* withstands desiccation in PIF and thrives in reconstituted PIF, especially when PIF is temperature-abused (Breeuwer et al., 2003; Riedel and Lehner, 2007; Osaili et al., 2009). In response, medical and health professionals had been cautioned regarding the use of PIF; however, *C. sakazakii* infections in neonatal units are not solely due to consumption of contaminated PIF (Jason, 2012). For example, *C. sakazakii* has been reported in infants exclusively breastfed (Hurrell et al., 2009b; Broge and Lee, 2013; Ravisankar et al., 2014). Another concern is the frequency with which nasogastric tubes are used to deliver enteral nutrition in premature neonates (Axelrod et al., 2006). A surveillance study reported that several species of *Enterobacteriaceae*, including a single *C. sakazakii* isolate, were recovered from used nasogastric enteral feeding tubes (Hurrell et al., 2009b). These researchers cautioned that microbial biofilms on nasogastric enteral feeding tubes might serve as a continuous inoculum during bolus feedings while the tube is in place. A simple solution may be to switch from indwelling nasogastric tubes to insertion of a nasogastric tube at each feeding; however, the comfort of the neonate and associated economic costs must be considered (Symington et al., 1995). A multifactorial approach to protecting neonates from microbial infections associated with feedings is needed, including identification of the mechanisms *C. sakazakii* uses during biofilm formation and gastrointestinal colonization.

Many bacteria, especially pathogens, have developed elaborate mechanisms to permit attachment to and formation of dense sessile mono- or polymicrobial aggregates on biotic and abiotic surfaces (Costerton et al., 1987; An and Friedman, 1998; Schluter et al., 2015). Following this initial attachment, bacterial aggregates can cooperatively form biofilms, thereby increasing their chance of survival. Herein, the formation of monospecies aggregates is referred to as autoaggregation. Autoaggregation is common in the *Enterobacteriaceae* family, including *Escherichia coli* (Girón et al., 1991; Czeczulin et al., 1997; Prigent-Combaret et al., 2000; Schembri et al., 2001; Sherlock et al., 2005; Girard et al., 2010; Nakao et al., 2012), *Salmonella* spp. (Collinson et al., 1993), *Klebsiella pneumoniae* (Favre-Bonte et al., 1995), *Edwardsiella tarda* (Gao et al., 2015), *Citrobacter freundii* (Bai et al., 2012), *Yersina pestis* (Vadyvaloo et al., 2015), and *Proteus mirabilis* (Rocha et al., 2007; Alamuri et al., 2010), and often occurs via self-recognizing cell-surface appendages. Autoaggregation is mediated by adhesins (Sherlock et al., 2005; Alamuri et al., 2010; Girard et al., 2010; Abdel-Nour et al., 2014; Arenas et al., 2015; Wang et al., 2015) and other cell-surface molecules, such as surface-associated proteins (Prigent-Combaret et al., 2000; Gao et al., 2015), pili (Girón et al., 1991), fimbriae (Nataro et al., 1992; Collinson et al., 1993; Czeczulin et al., 1997; Schembri et al., 2001), flagella (Sjoblad et al., 1985; Näther et al., 2006), and lipopolysaccharides (Nakao et al., 2012; Wang et al., 2012). Using microscopy, supercoiling between neighboring microorganisms promoted by pili in *Escherichia coli* was observed (Girón et al., 1991). Additionally, autoaggregation was mediated by flagella in *Pseudomonas marina* (Sjoblad et al., 1985) and *Pyrococcus furiosus* (Näther et al., 2006). The gastrointestinal colonization of two *Escherichia coli* pathotypes occurs via different fully characterized mechanisms of autoaggregation. Bundle-forming fimbriae (AAF/I and AAF/II) in enteroaggregative *E. coli* promote autoaggregation and biofilm formation along the intestinal surface (Nataro et al., 1992; Czeczulin et al., 1997), whereas enteropathogenic *E. coli* adheres to the intestinal surface via interactions between Intimin and Tir (Donnenberg and Kaper, 1991) and establishes three-dimensional microcolonies using bundle-forming pili (Girón et al., 1991). Although autoaggregation was observed in some *C. sakazakii* strains (Lehner et al., 2005; Wang et al., 2012; Hu et al., 2015), the extracellular factor mediating autoaggregation in *C. sakazakii* and its biological function were not described.

The role of bacterial flagella in motility and bacterial chemotaxis is well characterized (Sourjik and Wingreen, 2012), but motility is not its sole biological function. Bacterial flagella contribute to the virulence of bacterial pathogens, including adhesion, microcolony formation, invasion, and biofilm formation, as reviewed by others (Haiko and Westerlund-Wikström, 2013). Unlike other *Enterobacteriaceae*, the contribution of *C. sakazakii*'s flagellum to its virulence has received little attention. The flagella of *C. sakazakii* ES5 are required for adhesion to Caco-2 monolayers and biofilm formation to microtiter plates (Hartmann et al., 2010). Herein, we describe the role played by the bacterial flagella in the autoaggregation of *C. sakazakii* ATCC 29544 (CS29544). A collection of gene knockout and complementation strains revealed that structurally intact FliC containing filaments were required for autoaggregation. Additionally, we provide evidence to suggest that direct interactions between neighboring filaments promote autoaggregation of liquid CS29544 cultures.

## Materials and methods

### Bacterial strains and growth conditions

CS29544 was cultured in brain heart infusion (BHI) broth (Becton Dickinson), pH 7.38 at 37°C overnight aerobically with agitation (250 rpm) unless specified. CS29544 was enumerated and spread-plated on BHI agar plates following serial dilution in 1 × phosphate buffered saline (PBS; Dulbecco's Formula), pH 7.4. *Escherichia coli* was cultured in lysogeny broth (LB, Miller's formula) at 37°C overnight aerobically with agitation (250 rpm) unless specified. When necessary, ampicillin or chloramphenicol were added to BHI or LB at a final concentration of 100 and 35 μg/mL, respectively. To test for motility, CS29544 were grown on 0.4% agar composed of 3 g/L beef extract, 10 g/L Bacto peptone, 5 g/L sodium chloride (BPN) supplemented with 1% of 2, 3, 5-triphenyltetrazolium chloride (redox indicator) or observed microscopically by wet mount.

### Autoaggregation assays

Stationary phase CS29544 cultures, grown in 10 mL BHI at 37°C, were held statically at room temperature (~25°C) for 6 h to allow autoaggregation. The change in optical density at 600 nm was gently measured at 30 min intervals for 2 h followed by 1 h intervals until 6 h. Autoaggregation was reported as the maximum percent autoaggregation. Typically the endpoint was used, and calculated by Equation (1).

(1) $$Percent\;of\;autoaggregation\;=\;\frac{1-OD_{time\;point}}{OD_{initial}}\;*\;100$$

Several additional autoaggregation assays were conducted with modifications after the growth of CS29544 in 10 mL BHI, including static incubation at different temperatures (4 and 37°C), the addition of 50 mM EDTA or PBS, and before and after blending at “whip” speed for 30 s (BL113SG; Black and Decker). Furthermore, autoaggregation assays were completed with CS29544 following growth in 10 mL BHI at different pH values (pH = 4, 5, 6, 7.38, and 8) or incubated anaerobically (90% N_2_, 5% CO_2_, 5% H_2_). Finally, autoaggregation assays were run with CS29544 following growth (10 mL) in different media, including Miller and Lennox LB formulations (LB10 and LB5, respectively), tryptic soy broth (TSB), and BPN broth.

Stationary phase CS29544 and nonautoaggregating clonal variant (CV) cultures (described below) grown in BHI were mounted and held statically at room temperature for 1 h to allow autoaggregation. Still images were taken every 10 s for a total of 1 h by a stationary DSLR camera (Rebel T2i; Canon) with an intervalometer. Images (360 frames) were stitched together to create a video file with 24 frames per second. An additional time lapse video was constructed as previously described with the CS29544 and flagellum competition assays (described below) with still images taken every 20 s for a total of 6 h. Images (1,080 frames) were stitched together to create a video file with 72 frames per second.

### Isolation of nonautoaggregating CS29544 clonal variants

Stationary phase CS29544 cultures autoaggregated for 2 h. Then, two separate 100 μL (1%; v/v) aliquots, one from the top fraction of the CS29544 culture and one from the bottom fraction (autoaggregating control) were passed into two fresh tubes of 10 mL BHI broth and incubated as described above. Successive passages following autoaggregation continued until autoaggregation was arrested. Two independent nonautoaggregating variants were isolated and characterized.

### DNA extraction and whole-genome sequencing

Genomic DNA was isolated from CS29544 and CVs using the UltraClean® Microbial DNA Isolation Kit (MoBio Laboratories) according to manufacturer's instructions. High-quality genomic DNA libraries were prepared and sequenced using the Illumina platform by the DNA Services group affiliated with the Roy J. Carver Biotechnology Center at the University of Illinois-Urbana Champaign. Paired-end libraries of CS29544 and CVs were prepared with the TruSeq Genomic DNA Sample Prep Kit (length: 200–600 bp) and sequenced on a HiSeq2500 with the TruSeq SBS Sequencing Kit v1 producing a mean read length of 160 nt. An additional mate-pair library of CS29544 was prepared with the Nextera Mate Pair Library Sample Prep Kit (length: 3–8 kbp) and sequenced on a MiSeq V3 with the MiSeq 600-cycle Sequencing Kit v3 producing a mean read length of 300 nt. Paired-end reads were imported into CLC Genomics Workbench v7.5, and quality and adapter trimmed using default settings. Mate-pair reads were quality and adapter trimmed using Cutadapt (Martin, 2011) and an in-house Perl script provided by the Roy J. Carver Biotechnology Center. Processed mate-pair reads were imported into CLC Genomics Workbench and *de novo* assembled using default parameters, and only contigs larger than 1,000 bp were kept. The paired-end reads were mapped to the CS29544 *de novo* assembly and putative single nucleotide polymorphisms were identified (>90% frequency) using the Basic Variant Detection tool with default parameters confirmed by targeted Sanger sequencing using an ABI 3730XL capillary sequencer (Life Technologies).

### Construction of CS29544 gene knockout strains and complementation vector

Targeted gene disruptions (*flhA, fliG, motAB, fliC*, and *flaA*) were constructed in the wild-type CS29544 using the lambda Red recombinase system (Cherepanov and Wackernagel, 1995; Datsenko and Wanner, 2000). All bacterial strains, plasmids, and primers used in this study are listed in Tables 1, 2. Briefly, linear DNA fragments were amplified by PCR with pKD3 DNA using the target gene specific primer set (60 bp) and appropriate experimental conditions. CS29544 containing the pKD46 plasmid were grown in 10 mL of LB containing 10 μg/mL of ampicillin and 10 mM L-arabinose at 30°C overnight aerobically with agitation (250 rpm). CS29544 pKD46 electrocompetent cells were transformed with 500 ng of the purified linear DNA fragment. The FRT-Cm^r^-FRT cassette in the recombinant mutants was cured by transformation and subsequent removal of the temperature-sensitive flippase (FLP) recombinase helper plasmid (pCP20). The double gene knockout (*fliC* and *flaA*) was constructed as described above for the CS29544 Δ*flaA* strain. Gene disruptions were confirmed by junction fragment PCR using the appropriate primer sets and experimental conditions.

**Table 1:** **Bacterial strains and plasmids used in this study**.

**Strain or plasmid**	**Relevant characteristics or purpose**	**Internal number**	**Source or references**
***C. SAKAZAKII*** **STRAINS**			
CS29544	*Cronobacter sakazakii* ATCC 29544, wild-type	MJM187	ATCC
CS29544 pKD46	CS29544 harboring pKD46	MJM396	This study
*flhA*_CV (2.10)	CS29544 flhA nonautoaggregating variant	MJM387	This study
*fliG*_CV (3.6)	CS29544 fliG nonautoaggregating variant	MJM388	This study
Δf*lhA*	CS29544 *flhA*	MJM425	This study
Δ*fliG*	CS29544 *fliG*	MJM426	This study
Δ*motAB*	CS29544 *motAB*	MJM476	This study
Δ*fliC*	CS29544 *fliC*	MJM477	This study
Δ*flaA*	CS29544 *flaA*	MJM478	This study
Δ*flaA* pKD46	CS29544 *flaA* harboring pKD46	MJM480	This study
Δ*flaA*Δ*fliC*	CS29544 *flaAfliC*	MJM484	This study
Δ*fliC*/cfliC	CS29544 *fliC*/*fliC* complementation vector	MJM481	This study
Δ*flaA*Δ*fliC*/cfliC	CS29544 *flaAfliC* fliC complementation vector	MJM483	This study
***E. COLI*** **STRAINS**			
DH5α	pKD3 and pCP20 host strain	MJM363, MJM364	Gift from J. M. Slauch
MG1655	pKD46 host strain	MJM365	Gift from J. M. Slauch
Top10	cfliC host strain	MJM423	Invitrogen
**PLASMIDS**			
pCP20	*bla cat* cI857 λP_R_*flp*		Cherepanov and Wackernagel, 1995
pKD3	*bla* FRT *cat* FRT		Datsenko and Wanner, 2000
pKD46	*bla* P_BAD_ *gam bet exo*		Datsenko and Wanner, 2000
cfliC	1 kb fragment fliC native promoter and ORF cloned into pET-11a	pMJM49	This study

**Table 2:** **Primer sequences used to create and confirm gene knockouts**.

**Locus**	**Primers**	**Sequence (5′–3′)**
cat	c2_F	GATCTTCCGTCACAGGTAGG
	c1_R	TTATACGCAAGGCGACAAGG
flhA	flhA_ko_F	ATCACCAAGGGTGCGGGGCGTATCGCGGAAGTGGGCGCGC
	flhA_ko_R	GATAGGCTTCACCGCTGCCGATTTCCACGCCTTTCATCAG
	flhA_flank_F	GAAATCGGTCAGCAGATCC
	flhA_flank_R	CAGACCACGACCGTCATCAC
fliG	fliG_ko_F	TGACCATCGGTGAAGACCGCGCGGCGGAGGTGTTCAAACA
	fliG_ko_R	ATAGCTTTCTGTTCGTTTTCCACCTGAGACAGACGCACCG
	fliG_flank_F	AGCGTAAAGAAGTGGAAGAG
	fliG_flank_R	CGTCGACTTCTTCGCTTTC
motAB	motAB_ko_F	TGAACATCCTCGCCATAGCCAACAGCGGAAGGATGATGTC
	motAB_ko_F	AAATGTCTGATAAAAATCGCTAATATCCATACTCACGCTA
	motAB_flank_F	CGTAAACTTTCGCGAGATGCTG
	motAB_flank_R	CGCGGAATATGGCATTTAGCTG
fliC	fliC_ko_F	CAACCTGAACAAATCTCAGTCTGCTCTGGGCACTGCTATC
	fliC_ko_F	GAAAGCTTGAGCGTCTATTAACGCAGCAGGGACAGCATGG
	fliC_flank_F	ATGCAGACGCAGGCTATTGAG
	fliC_flank_R	TCCGGCTATATCTGTCGCAAC
flaA	flaA_ko_F	GCCACGCCGGTTGAGTCAGCGTCCGGGCAGGCGCGTGACG
	flaA_ko_F	GCCTTGTTAACGACTCTCCGATGTGCGAAACGGCAACCCT
	flaA_flank_F	CTTCCCGCTGACCATTTC
	flaA_flank_R	AACGTGCCGTCCATATCC

A *fliC* complementation vector was constructed by GenScript. Briefly, a 1,011 bp sequence, containing the *fliC* coding sequence and native promoter, was obtained from the publically available CS29544 genome (NCBI Reference Sequence: NZ_CP011047.1). The entire DNA fragment was synthesized and cloned into the pET-11a vector with the restriction enzymes *Bgl*II and *Bam*HI. The cfliC vector was electroporated into *E. coli* Top10 and subsequently electroporated into the CS29544 Δ*fliC* and Δ*flaA*Δ*fliC* strains using LB broth. Putative complements were grown in BHI or on motility agar plates supplemented with 50 μg/mL ampicillin. Restoration of wild-type function was assessed by autoaggregation assays, motility assays, microscopy, and flagella harvest as detailed above and below.

### Flagella staining and microscopy

The presence of extracellular flagella of CS29544, gene knockout, and complementation strains were determined by a combination of imaging techniques. Log or stationary phase CS29544, gene knockout, and complementation cultures were stained using a crystal violet-based flagella stain (Hardy Diagnostics) according to manufacturer's instructions. Stains were visualized using a light microscope at 1,000 × total magnification (BA210; Motic). Images were captured with a 2-megapixel Motic camera.

Several overnight colonies of CS29544, gene knockout, and complementation strains were gently lifted from BHI agar plates and suspended in phosphate buffered Karnovsky's fixative containing 2% glutaraldehyde and 2.5% paraformaldehyde. Transmission electron microscopy (TEM) was completed by the Beckman Institute's Microscopy Suite at the University of Illinois-Urbana Champaign. Briefly, the samples were stained with 2% uranyl acetate for 1 min and visualized using a CM200 LaB6 transmission electron microscope (FEI Co.). TEM was conducted at 120 kV and images were captured with a 2 k × 2 k digital camera (Tietz; Gauting; Germany). Several locations on the grids were examined, and the pictures were representative of the whole sample.

### Flagella harvest and filament protein identification, sequencing, and *In silico* analysis

The extracellular protein fraction of CS29544, gene knockout, and complementation strains was harvested by differential centrifugation (DePamphilis and Adler, 1971). Bacteria were cultured in two baffled flasks each containing 500 mL of BHI and incubated overnight at 37°C with agitation (250 rpm). Stationary phase cultures (1 L total) were centrifuged at 3,220 × g for 10 min at 4°C. Bacterial pellets were resuspended in a total of 250 mL 0.1 M Tris-HCl, pH 7.8, and blended at room temperature for 30 s at “whip” speed. Blended suspensions were centrifuged at 12,000 × g for 10 min at 4°C. The supernatant was further ultracentrifuged at 55,000 × g for 1 h at 4°C. Protein pellets were resuspended in a total of 1 mL 0.1 M Tris-HCl, pH 7.8, containing 50% glycerol (v/v, protein storage buffer) and stored at −20°C. Total protein was quantified with the Bradford Assay (BioRad Laboratories) and visualized with SDS-polyacrylamide gel electrophoresis. Typical flagellum protein recovery was 0.5–0.7 mg/mL from 1 L of cell mass (~10^12^ cells).

The putative FliC (28.9 kDa) band from CS29544 flagellum preparation was gel-excised and treated in-gel with trypsin (G-Bioscience) by the DNA Services group affiliated with the Roy J. Carver Biotechnology Center at the University of Illinois-Urbana Champaign. Protein was digested at a ratio of 1:20 (trypsin:protein) in 25 mM ammonium bicarbonate at 55°C for 30 min. Following lyophilization, peptides were analyzed by liquid chromatography-mass spectrometry. A total of 1–2 μg of digested peptides were loaded into a Dionex Ultimate 3000 RSLCnano connected directly to a Thermo LTQ-Velos-ETD Pro Mass Spectrometer (Thermo Fisher Scientific). Peptide were run on an Acclaim 300 C18 nano column (Thermo Fisher Scientific) using a gradient of 100% A (water + 0.1% formic acid) to 60% B (acetonitrile + 0.1% formic acid) at a flow rate of 300 nL/min. Raw data were collected by Xcalibur (Thermo Fisher Scientific) and processed with an in-house Mascot Distiller and Mascot Server (Matrix Science) and identified with the NCBInr database.

The secondary structure of the CS29544 FliC protein was predicted from the amino acid coding sequence (NCBI Reference Sequence: NZ_CP011047.1) using the Iterative Threading Assembly Refinement (I-TASSER) method (Yang and Zhang, 2015) with default parameters. The I-TASSER method is publically available at http://zhanglab.ccmb.med.umich.edu/I-TASSER/, accessed 10/15/2015. The theoretical secondary structures of FliC were visualized and modified using UCSF Chimera v.1.10.2 (Pettersen et al., 2004), publically available at https://www.cgl.ucsf.edu/chimera/. The hydrophobicity index of the primary FliC amino acid sequence was determined using the ProtScale tool from the ExPASy Bioinformatics Resource Portal (Gasteiger et al., 2005), publically available at http://www.expasy.org/, accessed 06/01/2016. The hydrophobicity index was calculated using the Kyte and Doolittle amino acid scale (Kyte and Doolittle, 1982) with a window size of 15 amino acids.

### CS29544 and flagellum competition assays

A 3 mL aliquot of stationary phase CS29544 culture was mixed with detached flagellar pieces at a concentration of 0.1, 1, 5, 10, or 20 μg/mL of total flagellum protein and autoaggregated for 6 h. Controls included adding 20 μg/mL of bovine serum albumin (BSA) or equal volume of protein storage buffer (no protein).

### CS29544 biofilm formation to polyvinyl chloride tubing

Polyvinyl chloride tubing (PVC; 0.318 cm outer diameter; 0.159 cm inner diameter; U.S. Plastic Corporation) was cut into 5 cm long pieces (external surface area ~5.15 cm^2^) with a sterile blade. The PVC tube pieces were disinfected and submerged in 70% ethanol for 10 min and aseptically dried. Two PVC tube pieces were aseptically transferred to a 15 mL centrifuge tube containing 10 mL of BHI supplemented with 50 μg/mL ampicillin for the Δ*fliC*/cfliC strain. Each experimental vessel was inoculated with 1% (v/v) stationary phase CS29544, Δ*motAB*, Δ*fliC*, and Δ*fliC*/cfliC cells and incubated vertically at 37°C aerobically with agitation (250 rpm) for 24 h. After incubation, each PVC tube piece was transferred with sterile forceps and washed three times in 5 mL PBS, pH 7.4. After washing, each PVC tube piece was placed into 30 mL PBS, pH 7.4, containing 3 g of autoclaved 0.1 mm diameter glass beads (Research Products International). Biofilms were subsequently disrupted by vortex at maximum speed for 1 min. Bacterial biofilm populations were enumerated by serial dilution (10^−1^–10^−3^) in 0.1% peptone water, spread-plated (0.1 mL in triplicate) on BHI agar plates, and incubated at 37°C overnight aerobically.

### Statistical analysis

Autoaggregation and biofilm formation assay results were from a minimum of three independent replicates. The differences between the mean maximum percent autoaggregation due to temperature, redox balance, pH, blending, and various media; the differences between biofilm formation due to the presence of FliC were determined with SAS® (Version 9.4; SAS Institute) using the generalized linear model. When statistical significance was observed (*P* < 0.05), a *post-hoc* mean separation was run using Tukey's Honest Significance Difference test which controlled for unequal sample sizes. All data satisfied the assumptions of normality and homogeneity of variance.

## Results

### Characterization of autoaggregation in CS29544

**Figure 1:**
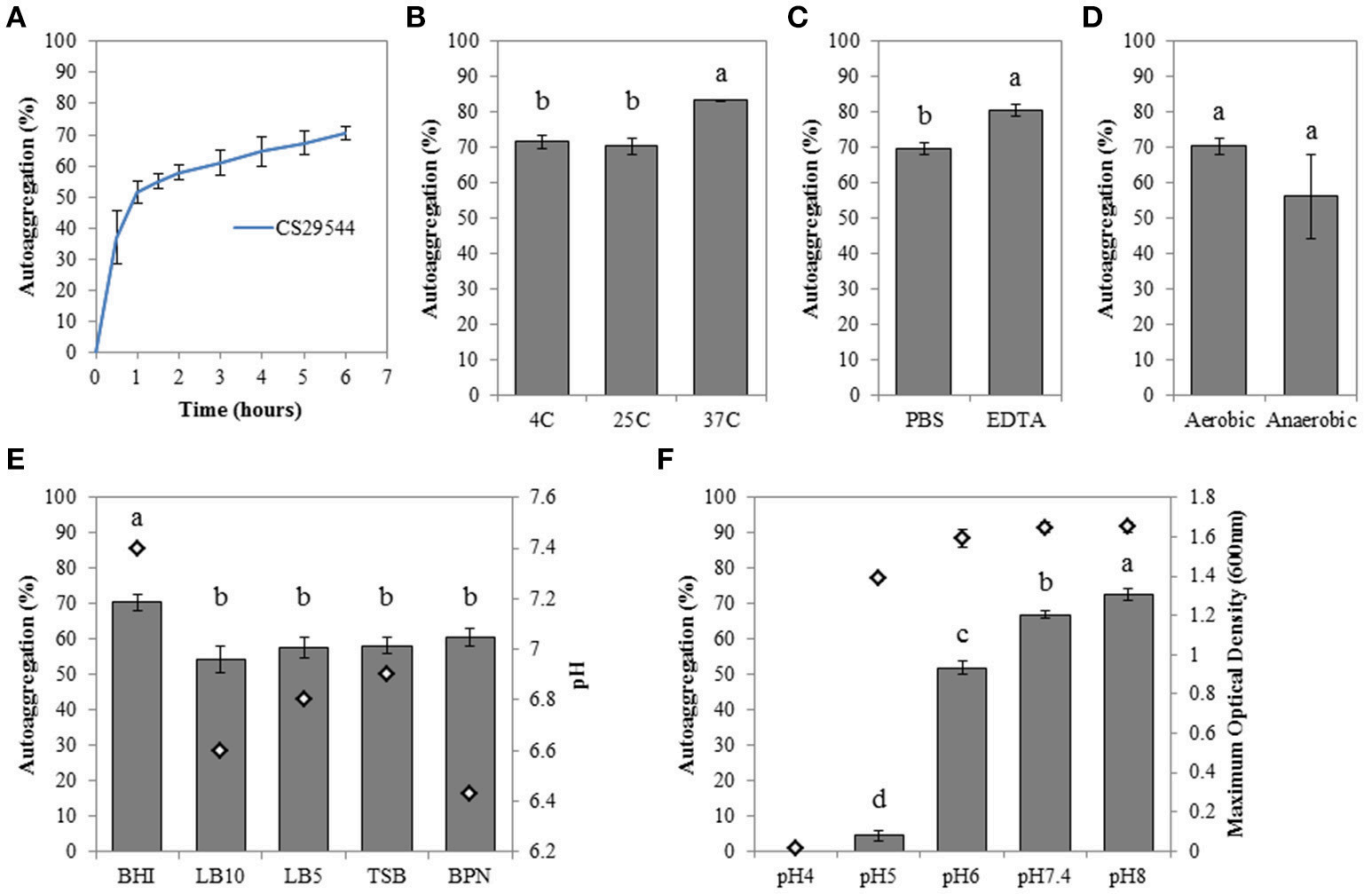
**Autoaggregation of stationary phase CS29544 cultures. (A)** Time course of autoaggregation in BHI broth over 6 h. Maximum percent autoaggregation **(B)** in BHI broth during static incubation at different temperatures; **(C)** in BHI broth in the presence of 50 mM EDTA or PBS; **(D)** in BHI broth following growth aerobically or anaerobically; **(E)** in a variety of growth media; LB10 = LB Miller's formula (10 g/L NaCL), LB5 = LB Lennox's formula (5 g/L NaCL), TSB = tryptic soy broth and BPN = motility broth, initial pH of growth media is reported for reference; **(F)** in BHI at different initial pH values. Maximum optical density (600 nm; open diamonds) is reported for reference. All experiments are mean ± standard error of three independent replicates. Values with no letters in common are significantly different (*P* < 0.05).

Stationary phase BHI cultures of CS29544 autoaggregated at 25°C within 30–60 min (Figure 1A). Following 6 h static incubation, the maximum mean autoaggregation percentage was 70.3 ± 2.2%. Various growth media and physiological conditions were tested to better understand CS29544 autoaggregation. Autoaggregation of BHI-grown cells at 37°C was significantly higher when statically incubated at 37°C than at 4 or 25°C (*P* = 0.0022; Figure 1B) or in the presence of EDTA (*P* = 0.0092; Figure 1C) compared to the PBS-added control. Although the maximum mean autoaggregation percentage was lower for cells grown in a reduced environment, the difference was not significant (*P* = 0.3003, Figure 1D). Autoaggregation following overnight growth in BHI was significantly higher than overnight growth in LB10, LB5; TSB, and BPN (*P* = 0.0203; Figure 1E). The difference in autoaggregation was not readily explained by the presence of salt, extracts, protein sources, or inclusion of phosphates but rather the initial pH of the media. Therefore, CS29544 was grown in BHI with varying initial pH values (range pH 4–8). CS29544 did not grow at pH 4, but its maximum growth was not affected by BHI from pH 5–8 (Figure 1F). As predicted, the maximum mean autoaggregation percentage decreased with decreasing pH (*P* < 0.0001; Figure 1F). The maximum mean autoaggregation percentage in BHI at an initial pH 6 was 51.9 ± 1.6%, which was slightly lower than the maximum mean autoaggregation percentage observed in the various media tested. During this initial characterization, an abolishment of autoaggregation was only achieved when grown in BHI at pH 5; however, this observation did not clearly point to a specific mechanism. Since the maximum mean autoaggregation percentage never reached 100%, we hypothesized that there might be a nonautoaggregating subpopulation of CS29544 mediated by an identifiable genetic variation.

### Nonautoaggregating CS29544 are a stable genetically distinct subpopulation

**Figure 2:**
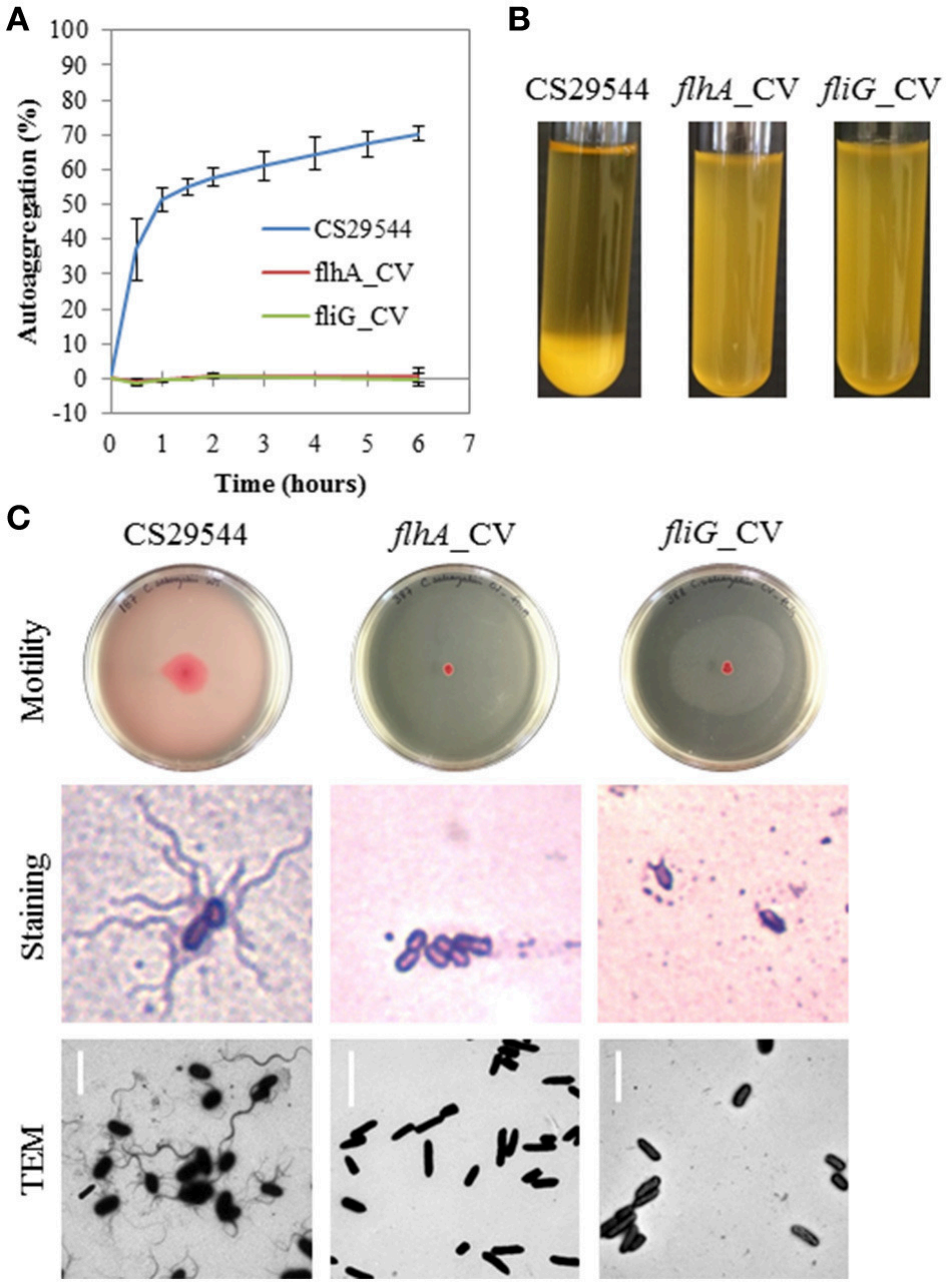
**Autoaggregation, motility and flagellation of nonautoaggregating CS29544. (A)** Time course of autoaggregation in BHI broth over 6 h. CS29544 (included for reference) and clonal variants: *flhA*_CV and *fliG*_CV. Experiment is mean ± standard error of three independent replicates. **(B)** Autoaggregation tube assays in BHI broth after static incubation for 6 h. **(C)** Motility agar plates centrally inoculated and imaged after 24 h, flagellation as detected by crystal violet staining at 1,000 × total magnification and TEM, bar = 4 μm.

Following five successive passages selecting against autoaggregating CS29544, we were able to isolate two independent nonautoaggregating CVs of CS29544 (Figures 2A,B; see Movie S1 in the Supplementary Material). *De novo* assembly of the mate-pair library preparation from wild-type CS29544 was used as the reference genome for comparative genomic analysis with the nonautoaggregating CVs (2.10 and 3.6) assemblies. One unique single nucleotide polymorphism was detected in each of the nonautoaggregating CV. Strain 2.10 contained a putative deletion of two consecutive base pairs (GC), while strain 3.6 contained a putative deletion of a single base pair (C). Further corroboration by Sanger sequencing revealed the two nonautoaggregating CVs contained frameshift mutations, and NCBI BLAST analysis revealed that these mutations were located in the open reading frames of two flagellum proteins, FlhA (2.10) and FliG (3.6). Comparison with the full length wild-type FlhA and FliG proteins (692 and 340 amino acids, respectively) revealed that the frameshift mutations results in truncated proteins (157 and 183 amino acids, respectively). Strains carrying these variant mutations are referred to as *flhA*_CV (2.10) and *fliG*_CV (3.6), respectively. Accordingly, we assessed the flagellation of the wild-type CS29544, *flhA*_CV, and *fliG*_CV with motility assays and microscopy (Figure 2C). Both the *flhA*_CV and *fliG*_CV strains were nonmotile and aflagellate by staining and TEM (Figure 2C). As a result, we constructed a variety of gene knockouts to determine if structurally intact and functional flagella were required for autoaggregation in CS29544.

### Flagellum structure, not function, is required for autoaggregation in CS29544

**Figure 3:**
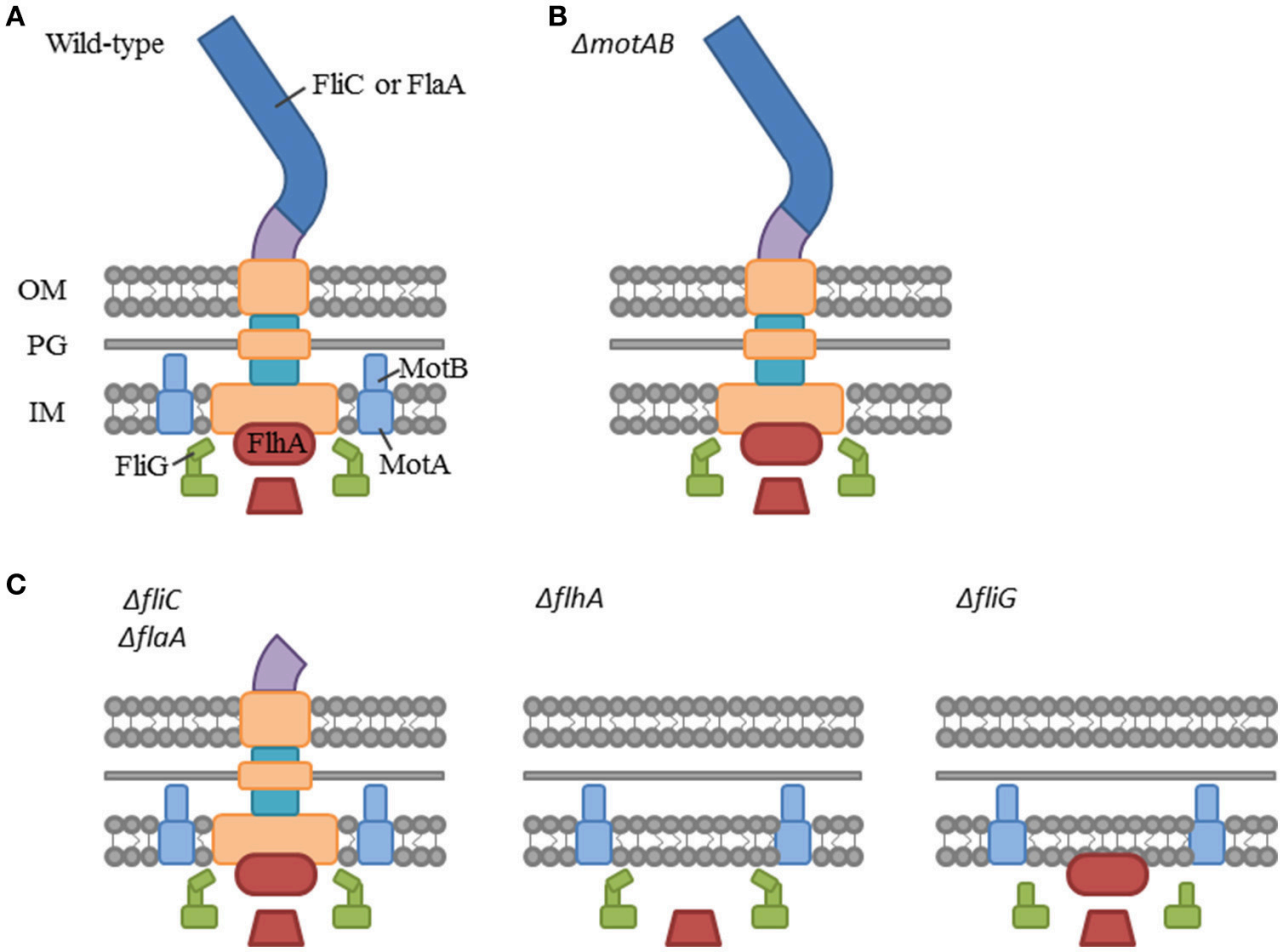
**Simplified diagram of the bacterial flagellum highlighting our gene knockout strategy. (A)** Bacterial flagellum labeled with gene knockout targets. Filament proteins: FliC and FlaA, Stator (motor) proteins: MotA and MotB, C-ring protein: FliG, and Exporter apparatus protein: FlhA. **(B)** Hypothesized flagellum structure in the functional mutant: Δ*motAB*. **(C)** Hypothesized flagellum structure in the structural mutants: Δ*fliC* and Δ*flaA*, Δ*flhA*, and Δ*fliG*.

Published *C. sakazakii* genomes, available from NCBI, have annotated some 40 genes related to their flagellum's structure, function, and regulation. Therefore, gene knockout strains (Table 1) were constructed to disrupt the structure and function of the CS29544 flagella (see Figures 3A–C for a simplified diagram of the bacterial flagella outlining our knockout strategy). Two basal body proteins, FlhA and FliG (same genes as the nonautoaggregating CVs) were targeted. Previously, FlhA truncation mutants in *Campylobacter jejuni* resulted in aflagellate cells lacking flagellar components past the inner membrane (Abrusci et al., 2013). FliG, along with FliN and FliM, forms the C ring of the basal body (Zhao et al., 2014). Proton-driven conformational changes in the MotA and MotB stator (motor) proteins (Kojima and Blair, 2001) directly interact with the C terminus of the FliG protein of the C ring to provide rotation to the flagella (Irikura et al., 1993; Lloyd et al., 1996). Although FliG is integral for the flagellum function, its necessity for assembly is disputed (Irikura et al., 1993; Lloyd et al., 1996). It was hypothesized that the disruption of *motAB* would render the cells nonmotile while retaining the structural components. Additionally, the disruption of *flhA* and *fliG* would block the early assembly of the flagella and the cells would be aflagellate. Based on the published CS29544 genome, CS29544 has redundant filament proteins; therefore, both FliC and FlaA single and double gene knockouts were constructed. Finally, the function of the flagella was disrupted by targeting the two motor proteins, MotA and MotB.

**Figure 4:**
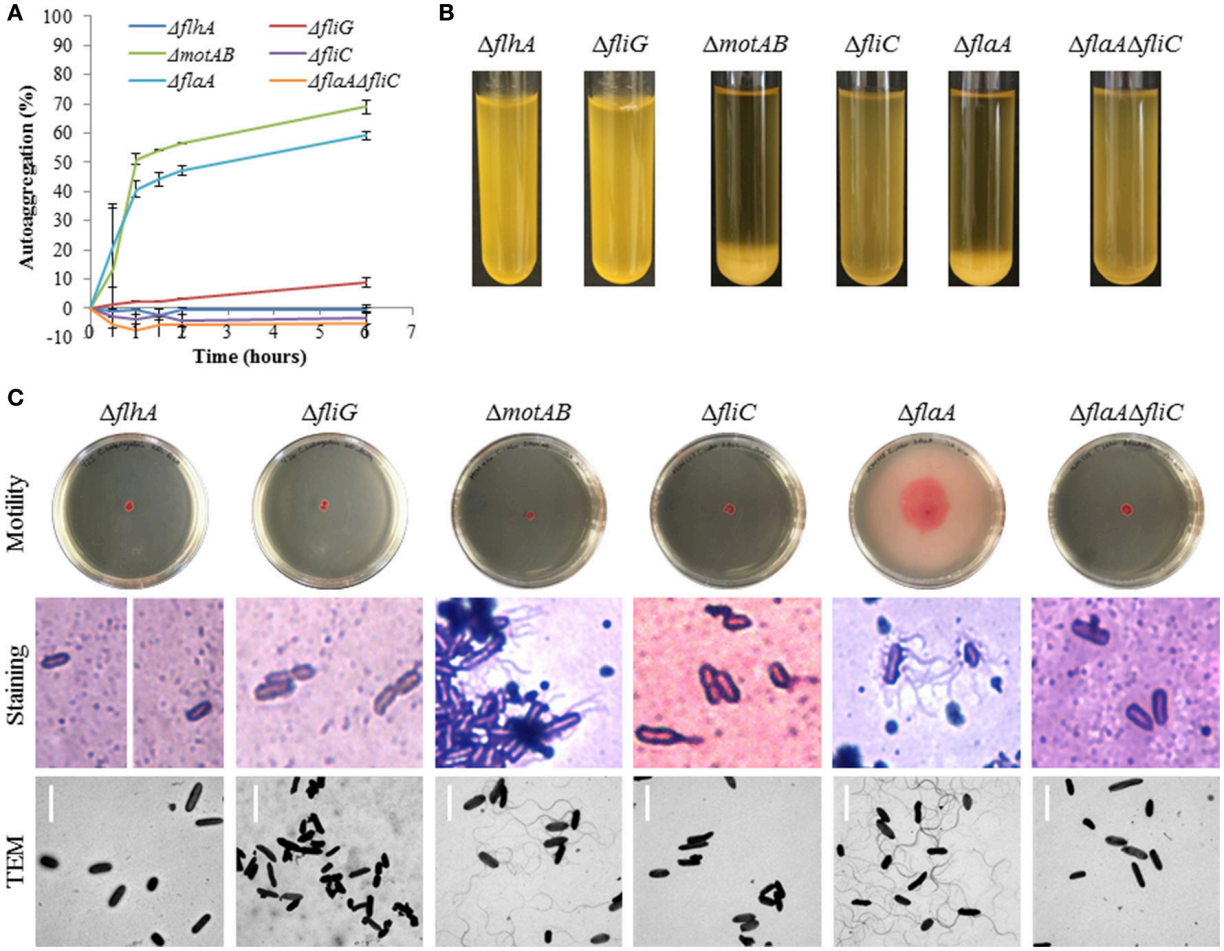
**Autoaggregation, motility, and flagellation of CS29544 and gene knockout strains. (A)** Time course of autoaggregation in BHI broth over 6 h. Gene knockouts: Δ*flhA*, Δ*fliG*, Δ*motAB*, Δ*fliC*, Δ*flaA*, Δ*flaA*Δ*fliC*. Experiment is mean ± standard error of three independent replicates. **(B)** Autoaggregation tube assays in BHI broth after static incubation for 6 h. **(C)** Motility agar plates centrally inoculated and imaged after 24 h, flagellation as detected by crystal violet staining at 1,000 × total magnification and TEM, bar = 4 μm.

**Figure 5:**
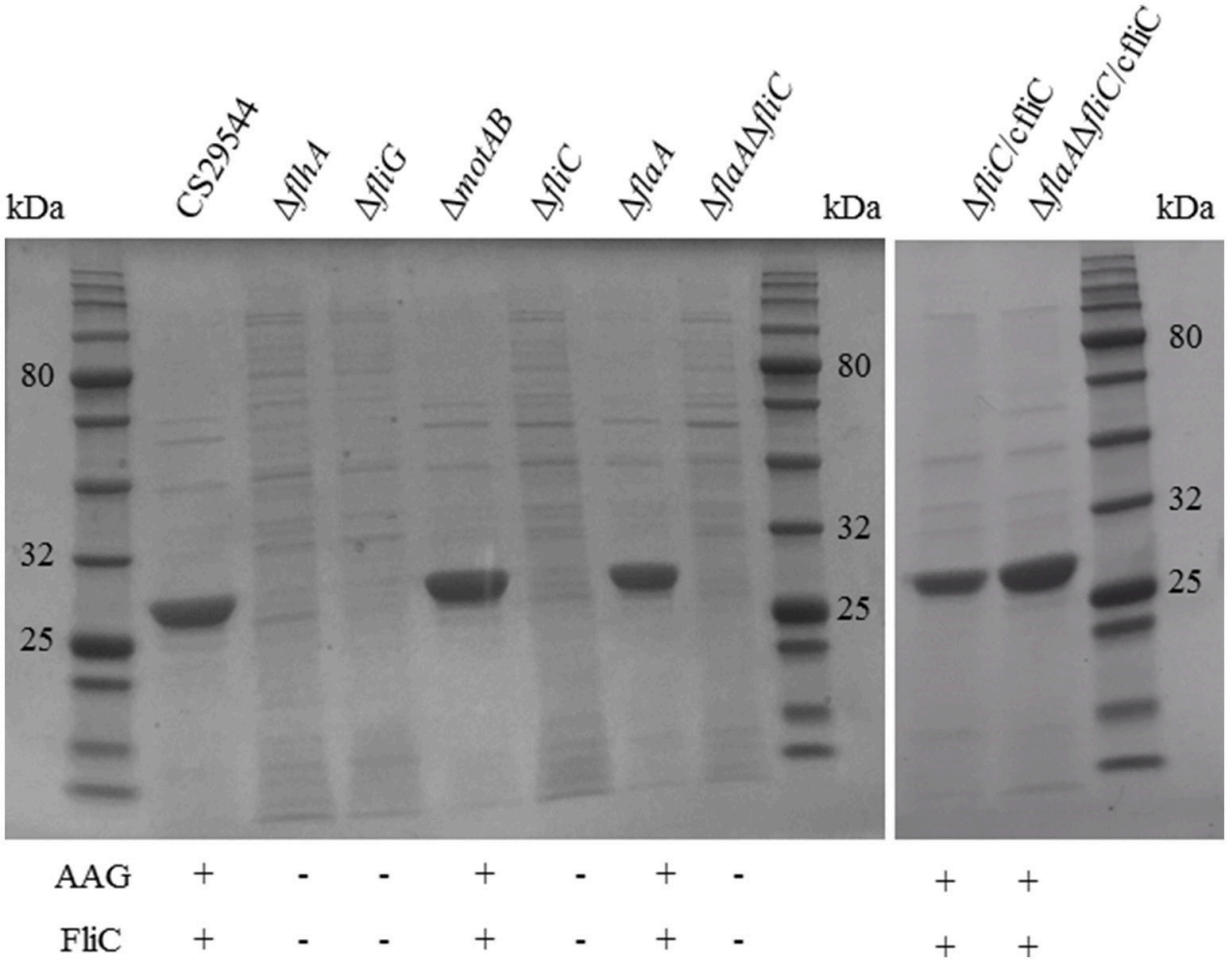
**Harvested filaments from of CS29544, gene knockout, and complementation strains**. Gene knockouts: Δ*flhA*, Δ*fliG*, Δ*motAB*, Δ*fliC*, Δ*flaA*, Δ*flaA*Δ*fliC*, and FliC complements: Δ*fliC*/cfliC and Δ*flaA*Δ*fliC*/cfliC. Each lane was loaded with ~2 μg of total protein. FliC expected size 28.9 kDa.

**Figure 6:**
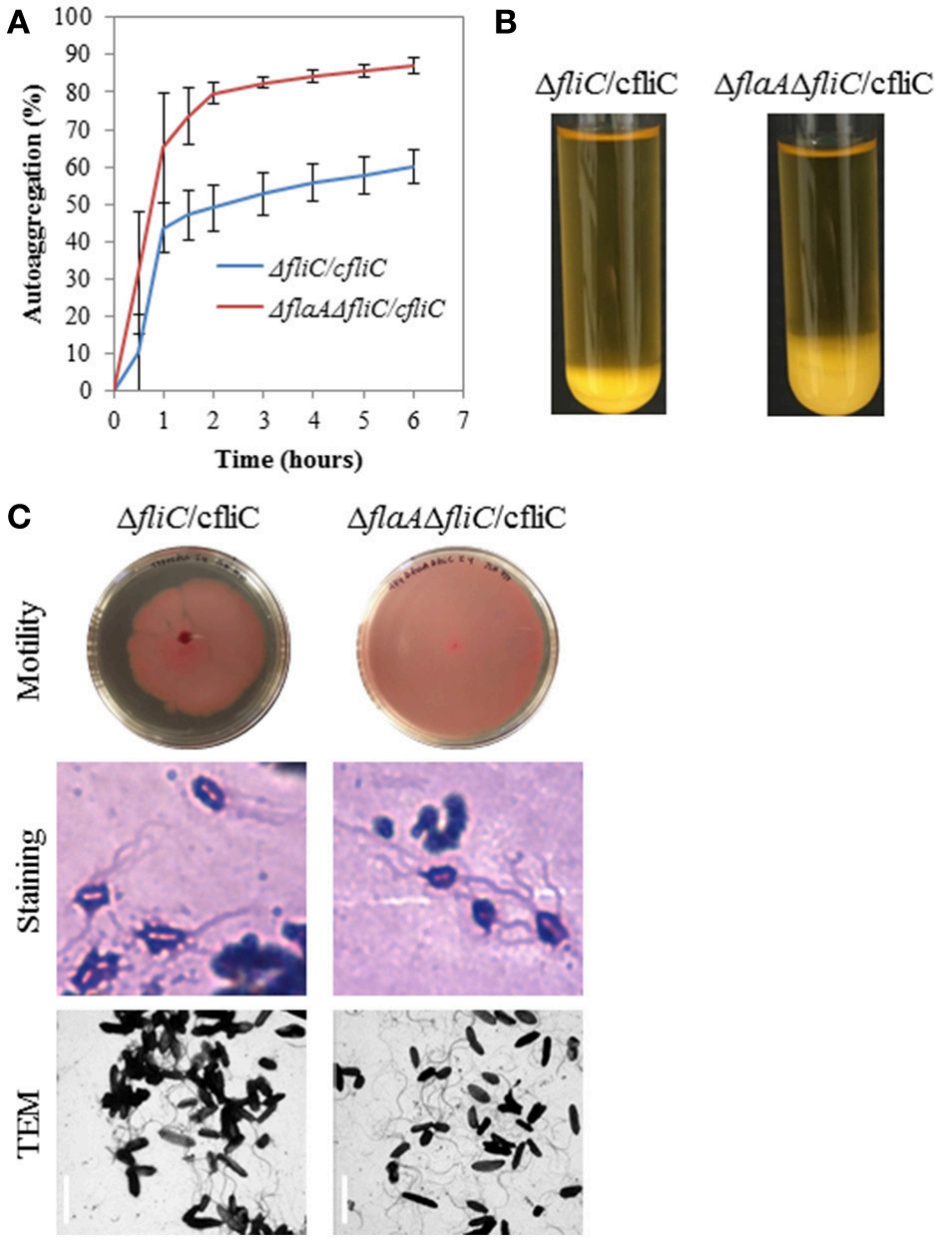
**Autoaggregation, motility, and flagellation of FliC complementation strains. (A)** Time course of autoaggregation in BHI broth over 6 h. FliC complements: Δ*fliC*/cfliC and Δ*flaA*Δ*fliC*/cfliC. Experiment is mean ± standard error of three independent replicates. **(B)** Autoaggregation tube assays in BHI broth after static incubation for 6 h. **(C)** Motility agar plates centrally inoculated and imaged after 24 h, flagellation as detected by crystal violet staining at 1,000 × total magnification and TEM, bar = 4 μm.

The Δ*flhA*, Δ*fliG*, Δ*fliC*, and Δ*flaA*Δ*fliC* strains did not autoaggregate and were aflagellate by staining and TEM and nonmotile (Figures 4A–C). Conversely, the Δ*motAB* and Δ*flaA* strains remained autoaggregative and had visible flagella by staining and TEM (Figures 4A–C). These two strains differed in their motility: Δ*flaA* was motile while Δ*motAB* was not motile. Therefore, we concluded that motility was not required for autoaggregation in CS29544. Based on the phenotypes of the structural gene knockouts, we hypothesized that filaments composed of FliC, not FlaA, were required for autoaggregation. Upon mechanical removal of the filaments, only the autoaggregating CS29544, Δ*motAB*, and Δ*flaA* strains possessed a dense 28.9 kDa protein band (Figure 5). The band was confirmed as FliC by a total of five peptides with individual ion scores of 44 and a Mascot ion score of 1,191 (data not shown). Furthermore, the Δ*fliC* strain did not contain a 50.1 kDa band, which is the predicted size for FlaA. Autoaggregation phenotype, motility, and flagellation were restored in the Δ*fliC*/cfliC and Δ*flaA*Δ*fliC*/cfliC complementation strains (Figures 6A–C). Finally, we confirmed a loss of autoaggregation in CS29544 after mechanical removal of flagella from 79.4 ± 4.4% to 2.2 ± 2.2% with no reduction in cellular viability (data not shown).

### Flagella-mediated autoaggregation occurs by flagellum-flagellum interactions

**Figure 7:**
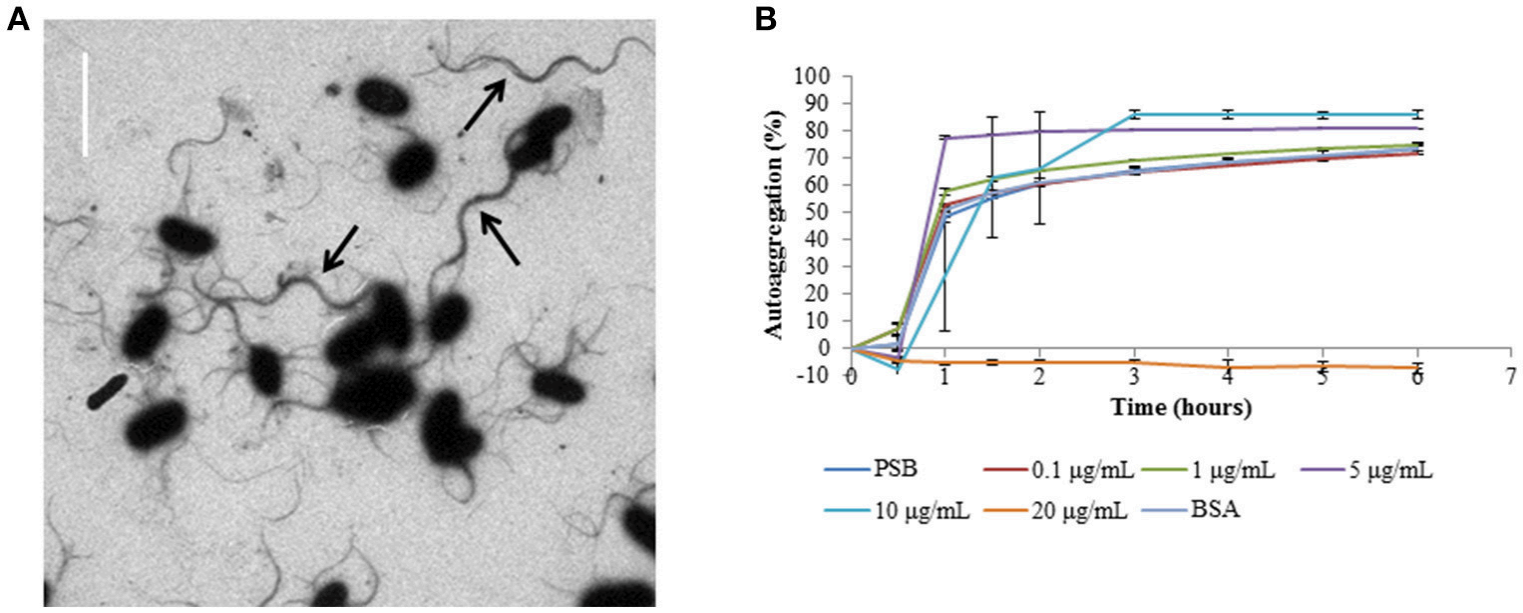
**Autoaggregation competition assays with harvested filaments from CS29544. (A)** CS29544 wild-type flagella TEM image, bar = 4 μm. Black arrows indicate bundles of flagella. **(B)** Time course of autoaggregation in BHI broth over 6 h. Stationary phase CS29544 cultures combined with harvested FliC filaments (0.1–20 μg/mL), bovine serum albumin (BSA, 20 μg/mL) and PSB (no protein control). Experiment is mean ± standard error of three independent replicates.

Visual analysis of the CS29544 TEM image revealed supercoiled flagella linking several bacteria together in a cluster (Figure 7A). Therefore, it was hypothesized that autoaggregation in CS29544 proceeds as flagella from one cell becomes entangled with the flagella from neighboring cells. To test this hypothesis, mechanically detached flagella pieces were added to stationary phase CS29544 cultures. Autoaggregation of CS29544 was not affected by adding 0.1 or 1.0 μg/mL of detached flagella, 20 μg/mL BSA, or the no-protein control (PSB; Figure 7B). Autoaggregation was prevented when 20 μg/mL of detached flagella were added. The addition of detached flagella altered the manner by which the CS29544 autoaggregated. The controls autoaggregated as before by forming small cell flocs that settled to the bottom of the tube. In the presence of 5 and 10 μg/mL of detached flagellum flocs did not form, rather a large mass of cells settled gradually to the bottom of the tube (see Movie S2 in the Supplementary Material).

**Figure 8:**
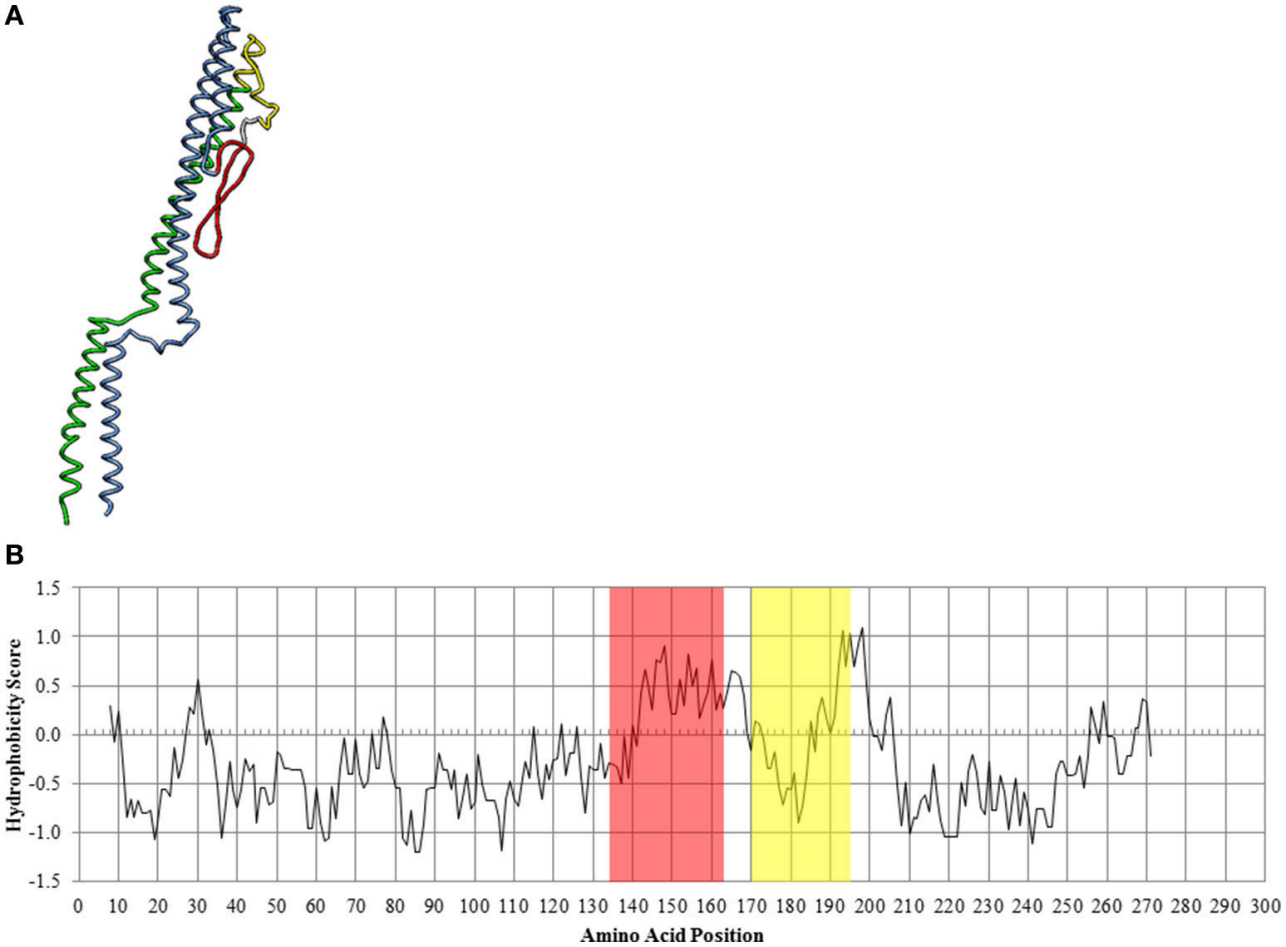
***In silico* analysis of the CS29544 FliC protein. (A)** Theoretical secondary structure of FliC from I-TASSER. Conserved regions: N terminus in blue, C terminus in green. Hypervariable region: hydrophobic peak in red, hydrophobic valley in yellow. **(B)** Hydrophobicity index along the linear amino acid sequence of FliC. Hydrophobic peak highlighted in red, hydrophobic valley highlighted in yellow.

The predicted secondary structure of the CS29544 FliC protein was analogous to homologous flagellins with a highly conserved flagellin N and C termini linked by a hypervariable region (Figure 8A). Analysis of the hydrophobicity scores of the linear amino acid sequence revealed a peak followed by a valley in hydrophobicity (Figure 8B) located in the hypervariable region of the CS29544 FliC. The bacterial filament is composed of several thousand flagellin proteins with the conserved regions stacked laterally. The hypervariable region is externally exposed and able to interact with its environment. We hypothesize that the hypervariable hydrophobic peaks and valleys of neighboring cells interact laterally to promote autoaggregation in CS29544. Further work will be required to test this hypothesis.

### Biofilm formation on PVC is not mediated by flagella

**Figure 9:**
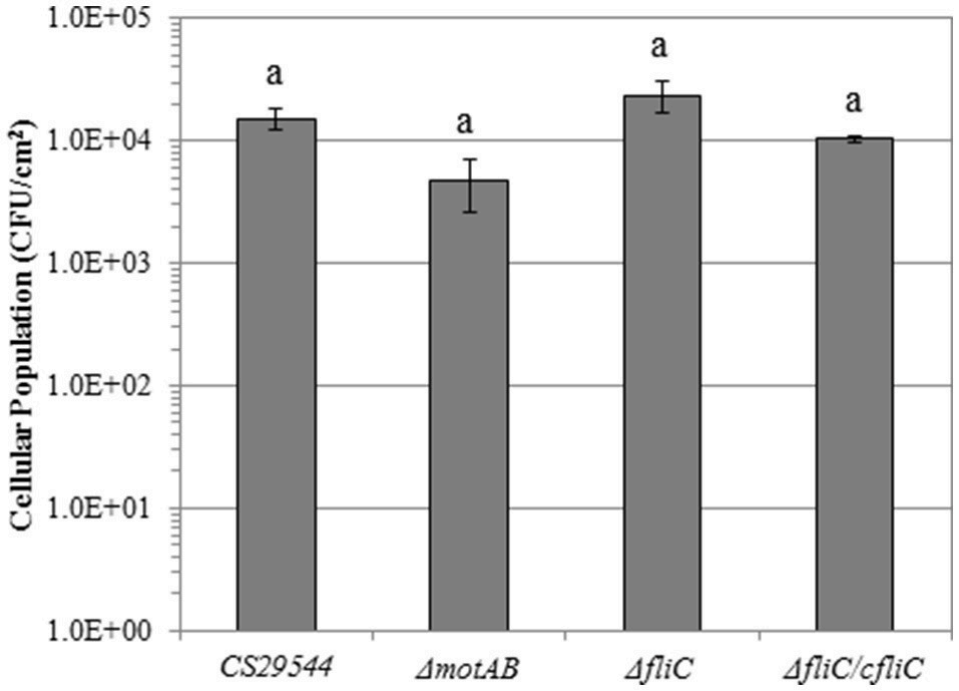
**Biofilm formation on polyvinyl chloride of CS29544 and select gene knockout and complementation strains**. Experiment is mean ± standard error of three independent replicates. Gene knockouts: Δ*motAB* and Δ*fliC* and FliC complement: Δ*fliC* /fliC. Values with no letters in common are significantly different (*P* < 0.05).

The total cellular population of wild-type CS29544 biofilms on PVC was 1.5 × 10^4^ ± 3.0 × 10^3^ CFU/cm^2^. The total cellular populations of Δ*motAB*, Δ*fliC*, and Δ*fliC*/cfliC on PVC biofilms were 4.8 × 10^3^ ± 2.2 × 10^3^, 2.4 × 10^4^ ± 7.0 × 10^3^, and 1.0 × 10^4^ ± 6.5 × 10^2^ CFU/cm^2^, respectively. CS29544 biofilm formation on PVC was not mediated by FliC under the tested conditions (*P* = 0.0928; Figure 9).

## Discussion

Although autoaggregation was demonstrated in *C. sakazakii* ATCC BAA_894 (Wang et al., 2012; Hu et al., 2015), the molecular basis for autoaggregation was not described. To understand the genetic determinants of autoaggregation in CS29544, a set of structural and functional flagellar mutants were constructed. These flagellar mutants revealed the requirement of FliC containing filaments in the autoaggregation of CS29544. Additionally, these results suggest an additional biological function for the CS29544 flagellum beyond motility.

Protein-protein interactions, such as flagella-mediated autoaggregation, may require specific environmental conditions. Previously, protein-protein aggregation in bacteria was influenced by altering the growth media (Girón et al., 1991), sodium chloride concentrations (Girard et al., 2010), pH (Sherlock et al., 2005; Alamuri et al., 2010), or the presence of divalent cations (Sjoblad et al., 1985; Abdel-Nour et al., 2014). In the present study, autoaggregation assays were conducted to identify nutritional dependencies and provide insights into potential mechanisms. Different growth media containing various nutrient extracts, protein sources, salts (NaCl and phosphates), and carbohydrates (dextrose) were tested. Additionally, autoaggregation was observed under a variety of temperatures, redox potentials, and pH values. Wild-type CS29544 was flagellated and highly motile under all tested growth conditions, except growth in BHI at pH 5. In hindsight, it is not surprising that autoaggregation in CS29544 had only minimal nutritional or conditional dependencies, even though flagellar expression is a tightly regulated system that quickly responds to changes in the surrounding bacterium environment (Osterman et al., 2015). Under favorable environmental conditions, such as nutrient-dense media, motility may be arrested following the downregulation of flagellar genes. However, nonmotile bacteria do not immediately shed their structurally intact flagella and these flagella can participate in other biological functions. Although abolishment of autoaggregation in CS29544 was observed in structural mutants (Δ*flhA*, Δ*fliG*, Δ*fliC*, and Δ*flaA*Δ*fliC*), autoaggregation was not affected in the functional mutant (Δ*motAB*) which retained the structural components. These results suggest that autoaggregation in CS29544 can serve as an additional biological function for the CS29544 flagellum under environmental conditions that favor the downregulation of motility but not the loss of structure. Of note, many other examples of autoaggregation in *Enterobacteriaceae* involve pili and fimbriae (Girón et al., 1991; Nataro et al., 1992; Collinson et al., 1993; Czeczulin et al., 1997; Schembri et al., 2001). While CS29544 has putative pilus and fimbrial genes, the present study did not identify a role for these structures in the autoaggregation of CS29544. Future studies are needed to investigate the role of pili and fimbriae in CS29544's pathogenicity.

The CS29544 genome encodes > 40 genes that are required for the assembly, function, and regulation of its flagellum. In this study, autoaggregation was only mediated by the loss of structural proteins, specifically, the lack of the FliC containing filament. Four structural mutants, two direct (Δ*fliC* and Δ*flaA*Δ*fliC*) and two indirect (Δ*flhA* and Δ*fliG*), resulted in aflagellate nonautoaggregating CS29544 cells. Since the extracellular filament, comprised of several thousand FliC monomers, extends several microns from the cell, it is physically able to promote cell-cell interactions. Upon close examination of wild-type CS29544 cells by TEM, neighboring cells appeared tethered by their filaments. Similar bundles were observed in *Escherichia coli* (Girón et al., 1991), *Pseudomonas marina* (Sjoblad et al., 1985)**, and *Pyrococcus furiosus* (Näther et al., 2006). Furthermore, flagella-mediated autoaggregation was disrupted in a dose-dependent manner by the addition of exogenous wild-type FliC filaments. Protein-protein interactions can be mediated by several factors, including ionic and hydrophobic bonds. As discussed above, only growth in BHI at pH 5 abolished flagella-mediated autoaggregation in CS29544 and no other nutritional or conditional dependencies were observed. Previously, TibA-mediated autoaggregation in an enterotoxigenic *E. coli* was affected by changes in pH (Sherlock et al., 2005). The authors speculated that TibA-mediated autoaggregation might be promoted by pH-mediated ionic bonds between charged amino acid side chains. It is tempting to conclude that flagella-mediated autoaggregation involves ionic bonding due to abolishment at pH 5; however, our observations do not support this conclusion. CS29544 cells grown in BHI at pH 5 were growth-impaired, had no visible flagella by staining, and were nonmotile by wet mount.

The CS29544 genome encodes two different flagellin proteins, *fliC* and *flaA*; however, only FliC monomers were incorporated into the harvested CS29544 filaments under the tested conditions. Consistent with this study, FliC is the sole *C. sakazakii* flagellin protein reported in the literature (Proudy et al., 2008; Cruz-Córdova et al., 2012). FliC flagellin proteins and their homologs have highly conserved N and C termini connected by a hypervariable region. The conserved domains of several flagellin proteins self-assemble and form the internal channel of the filament during elongation. The exposure of conserved domains to the bacterium's environment is limited and should not contribute to flagella-mediated autoaggregation. Conversely, the hypervariable region is invariably externally exposed and likely interacts with components of the bacterium's surroundings. As a result, our *in silico* methods were centered on the secondary structure and hydrophobicity of the hypervariable region. The entire CS29544 FliC flagellin protein is composed of 278 amino acids, of which 50 amino acids comprise the hypervariable region. Of note, the hypervariable region in the *C. sakazakii* FliC flagellin protein is far shorter than FliC flagellin proteins of related *Enterobacteriaceae* (Proudy et al., 2008). As seen in the predicted secondary structure, the hypervariable region is relaxed and spatially aligned with the conserved regions. Unfortunately, the predicted FliC secondary structure did not reveal any obvious structural contributions to flagella-mediated autoaggregation. Rather, alterations in hydrophobicity along the amino acid sequence illustrated the potential of hydrophobic interactions. It is hypothesized that there are, along the length of every filament, thousands of FliC monomers with alternating hydrophobic peaks and valleys (colored in red and yellow, respectively, in Figure 8A). The following hypothesis can be best exemplified by the dimerization of proteins by the leucine zipper motif. When filaments are in close proximity, it is hypothesized that these hydrophobic peaks and valleys interact to allow rapid and reversible supercoiling. Once a sufficient number of CS29544 cells are tethered together by their FliC filaments, autoaggregation by this mechanism proceeds. Further work is needed to test these hypotheses and to more precisely define which amino acids interact during flagella-mediated autoaggregation in CS29544.

*C. sakazakii* strains form biofilms on a variety of abiotic surfaces, including stainless steel (Iversen et al., 2004; Kim et al., 2006; Jung et al., 2013), silicon (Iversen et al., 2004), latex (Iversen et al., 2004), PVC (Lehner et al., 2005; Kim et al., 2006; Hurrell et al., 2009a), and polyurethane (Hurrell et al., 2009a). The latter two plastics are used for enteral feeding tubes and formation of *C. sakazakii* biofilms on these plastics is of concern. Hurrell et al. (2009b) isolated *C. sakazakii*, along with other pathogenic *Enterobacteriaceae* from used enteral feeding tubes. Biofilm formation on enteral feeding tubes is problematic for several reasons. First, enteral feeding tubes typically reside within an infant at body temperature (37°C) for several days (Mehall et al., 2002). Secondly, infant feeds are nutrient-dense and provide sufficient growth substrate for bacteria. Lastly, with every feeding, bacteria might dislodge from the biofilm and continuously inoculate the neonate (Mehall et al., 2002; Hurrell et al., 2009b). To determine the impact of flagella-mediated autoaggregation on *C. sakazakii* biofilm formation, the biofilm formation by the wild-type CS29544 was compared to the Δ*motAB*, Δ*fliC*, and Δ*fliC*/cfliC strains. To model *C. sakazakii* biofilm formation on neonatal enteral feeding tubes, flagella-mediated biofilm formation was tested in a nutrient-dense environment (BHI broth) at 37°C using PVC tubing. In the present study, the total cellular biofilm population on PVC tubing ranged from 3.7-log CFU/cm^2^ in the Δ*motAB* strain to 4.4-log CFU/cm^2^ in the Δ*fliC* strain. There was no significant difference in biofilm formation between the wild-type CS29544 and the Δ*motAB*, Δ*fliC*, and Δ*fliC*/cfliC strains under the tested conditions. The observed *C. sakazakii* population density was consistent with the mean biofilm population of 4.0-log CFU/cm^2^ on PVC tubing of five *C. sakazakii* strains grown in TSB at 12°C reported by Kim et al. (2006). Additionally, that study reported an approximate 1.5-log increase in the mean biofilm population (5.7-log CFU/cm^2^) on PVC tubing when *C. sakazakii* strains were grown in TSB at 25°C. Given that 27°C is the optimal temperature for *C. sakazakii* exopolysaccharide production, this result is not surprising. Admittedly, *C. sakazakii* biofilm formation due to differences in flagella-mediated autoaggregation phenotype was not robustly tested. To date, a single study has demonstrated the importance of *C. sakazakii* strain ES5 flagellum in biofilm formation and adhesion to microtiter plates (Hartmann et al., 2010). The data presented in the present study demonstrates that additional research into *C. sakazakii* flagella-mediated autoaggregation, biofilm formation, and gastrointestinal colonization is critically needed.

A significant shortcoming of this study is its limited scope. A single strain of *C. sakazakii* was characterized, and generalization to all *C. sakazakii* strains should be avoided. Currently, our collective understanding of *C. sakazakii* pathogenesis is insufficient. Several decades of work were completed to characterize the diverse pathotypes in *E. coli*, and it is tempting to speculate that *C. sakazakii* may have definable pathotypes of which flagella-mediated autoaggregation is important. Future studies should be designed to characterize flagella-mediated autoaggregation contributions to *C. sakazakii* pathogenesis *in vivo* with suitable animal models. Concurrently, autoaggregation, not necessarily flagella-mediated, should be characterized in several clinical, environmental, and laboratory *C. sakazakii* strains. The present study contributes much-needed knowledge to the *C. sakazakii* literature.

## Author contributions

JH conceived of the project, contributed to the design of the experimental methods, led the acquisition, analysis, and interpretation of the data other than experiments completed by the acknowledged collaborators, wrote initial and revised drafts of the manuscript, and approved the final manuscript submission. MM contributed to project design, selection of experimental methods, interpretation of data throughout the project, contributed to drafting and revising of the manuscript, approved the final manuscript submission. JH and MM agree to be accountable for the work detailed in the final manuscript submission.

## Funding

JH was supported by the Agnes and Bill Brown Fellowship in Microbiology from the University of Illinois-Urbana Champaign. This research received no direct financial support from any funding agency in the public, commercial, or not-for-profit sectors.

### Conflict of interest statement

The authors declare that the research was conducted in the absence of any commercial or financial relationships that could be construed as a potential conflict of interest.
